# Endophyte-Mediated Effects on the Growth and Physiology of *Achnatherum sibiricum* Are Conditional on Both N and P Availability

**DOI:** 10.1371/journal.pone.0048010

**Published:** 2012-11-20

**Authors:** Xia Li, Anzhi Ren, Rong Han, Lijia Yin, Maoying Wei, Yubao Gao

**Affiliations:** College of Life Sciences, Nankai University, Tianjin, P. R. China; University of Konstanz, Germany

## Abstract

The interaction of endophyte–grass associations are conditional on nitrogen (N) availability, but the reported responses of these associations to N are inconsistent. We hypothesized that this inconsistency is caused, at least in part, by phosphorus (P) availability. In this experiment, we compared the performance of endophyte-infected (EI) and endophyte-free (EF) *Achnatherum sibiricum* subjected to four treatments comprising a factorial combination of two levels of N (N+ vs. N−, i.e. N supply vs. N deficiency) and two levels of P (P+ vs. P−, i.e. P supply vs. P deficiency) availability. The results showed that *A. sibiricum–Neotyphodium* associations were conditional on both N and P availability, but more conditional on N than P. Under N+P− conditions, endophyte infection significantly improved acid phosphatase activity of EI plants, such that the biomass of EI plants was not affected by P deficiency (i.e. similar growth to N+P+ conditions), and resulted in more biomass in EI than EF plants. Under N−P+ conditions, biomass of both EI and EF decreased compared with N+P+; however, EI biomass decreased slowly by decreasing leaf N concentration more rapidly but allocating higher fractions of N to photosynthetic machinery compared with EF plants. This change of N allocation not only improved photosynthetic ability of EI plants but also significantly increased their biomass. Under N−P− conditions, EI plants allocated higher fractions of N to photosynthesis and had greater P concentrations in roots, but there was no significant difference in biomass between EI and EF plants. Our results support the hypothesis that endophyte–grass interactions are dependent on both N and P availability. However, we did not find a clear cost of endophyte infection in *A. sibiricum*.

## Introduction

Many grasses are infected by clavicipitaceous fungal endophytes that occur in aboveground plant tissues. Asexual endophytes live asymptomatically within the host tissues, receiving protection and nutrients, and are vertically transmitted to the next plant generation via host seeds. Based on numerous studies using tall fescue and perennial ryegrass in agronomic, fertilized soils, this symbiosis has been considered strongly mutualistic – mainly because endophyte infection may improve herbivore resistance of the host grasses due to production of alkaloids [1], and increase plant vigor and tolerance to a wide range of abiotic environmental conditions (e.g. drought) [2]–[3]. There is increasing evidence that the benefits from endophyte infection depend largely on the availability of other resources, in particular nutrients [4]. Resource limitation can increase the cost of supporting some endophytes [5]–[6], potentially changing the interaction from mutualism to parasitism or commensalism [7]. In fact, many of the studies that have found improved growth in endophyte-infected (EI) grasses were done under benign conditions of moderate to high soil nutrient availability [8]–[11].

Studies on endophyte-related responses of grasses to nutrient acquisition have focused on the influence of nitrogen (N), since this element is not only a constituent of alkaloids in infected plants but also one of the most important limiting resources for plant growth in nature. In the plant the photosynthetic apparatus is the largest sink of N [12]. Photosynthetic capacity and photosynthetic N use efficiency (PNUE) correlates strongly with N allocation to the photosynthetic machinery [13]. Small changes in N allocation can greatly influence light-saturated photosynthetic rate (P_max_) and PNUE, and therefore plant performance [14]–[16]. Consequently, leaf N allocation to photosynthesis is an important factor explaining differences in P_max_ and PNUE [14]. Published reports of the effects of endophyte infection on N use efficiency of grass-endophyte associations are inconsistent. Arachavaleta et al. [17] found beneficial effects of endophyte infection in tall fescue only at high N concentrations, and this result was further supported by our previous study in perennial ryegrass [11]. In contrast, Ravel et al. [18] found an advantage of EI plants over EF (endophyte-free) plants was greater at low N levels. It has been documented that increased N availability may also change the relative availability of other nutrients such as phosphorus (P) [19]–[20]. Therefore, we asked whether the inconsistent results are caused, in part, by other nutrients such as P.

Similar to N nutrition, P availability also influences ergot alkaloid production in EI grasses [21]. However, published reports of the effects of endophyte infection on P use efficiency of grass-endophyte associations are limited [22]. Malinowski et al. [23] found that EI tall fescue expressed an increased root absorption area through reduced root diameter and increased root hair length compared with the EF counterpart. The Fe^3+^ reducing activity on the root surface and total phenolic concentration in roots also increased dramatically in response to endophyte infection [24]. N addition may change the relative availability of P. On one hand, N addition could stimulate phosphatase activity of the root [25], which could potentially promote P uptake from bound-P. In fact, the production and excretion of acid phosphatase is considered to be one component of a plant phosphate-starvation rescue system [26]. On the other hand, a high N:P supply ratio could result in P starvation in the plant [27]. Populations previously limited by N can switch to limitation by P after receiving high N [19]–[20]. N:P stoichiometry in plant tissues, especially leaves, is related to growth strategy and can be an indicator of vegetation composition, functioning and nutrient limitation at the community level [28]. Until now, however, the effect of both N and P availability on grass–endophyte associations has received little attention.

Endophytic fungi not only occur in agronomic grasses but also in almost all habitats where grasses are common [29]. In our previous survey in the permanent grasslands of northern China, 25 of 41 species of grasses surveyed (61%) were infected by *Neotyphodium* endophytes [30]. However, most of the work for endophyte-plant interactions has been based upon endophyte-plant studies of two, economically important, artificially selected and non-native grass species [31]–[32]. Few studies exist to predict how wild plant–endophyte symbioses will respond to N and/or P availability, especially when the two elements are considered simultaneously. If infection competes with other plant functions for limiting nutrients, then infection may be more advantageous in environments with high soil nutrients [8], [33]. Alternatively, if systemic endophytes enhance nutrient uptake by the host [34], then infected plants may outcompete uninfected plants when nutrients are limited. Therefore, endophyte infection may have strong influences on plant community composition by altering the performance of host grasses relative to other species present in the community in response to different nutrient availability.

*Achnatherum sibiricum* (L.) Keng is a caespitose perennial grass that is widely distributed in northern China. After five years of continuous survey, Wei et al. [30] found that *A. sibiricum* was highly infected by *Neotyphodium* fungi, and there was little difference in infection rates among different geographic populations. Within the genus *Achnatherum* there are five sections, and *A. sibiricum* belongs to section Achnatheropsis (Tzvel.) N. S. Probatova. There are nine species in this section, including seven Asian and two American species [35]. Except for *A. sibiricum*, only two species, *A. inebrians* (Hance) Keng ex Tzvelev and *A. robustum* (Vasey) Barkworth, are reported to be infected by *Neotyphodium* endophytes. Both are notorious for their narcotic effects on livestock, and hence are known as ‘drunken horse grass’ and ‘sleepy grass’, respectively [36]–[37]. In contrast to *A. inebrians* and *A. robustum*, *A. sibiricum* has no obvious herbivore deterrence according to local records and our own observations. Here, we investigated whether the responses of *A. sibiricum* to endophyte infection depended on N and/or P availability. Specifically, we addressed the following questions: (1) does the endophyte improve performance of the native grass host? (2) does N and/or P availability affect the symbiosis-dependent benefits? If this is the case, (3) how does the nutrient availability affect the symbiosis-dependent benefits?

## Materials and Methods

### Ethics statement

No specific permissions were required since in this study we only collected a limited amount of seeds from a native grassland, and this grassland is not privately-owned or protected in any way. Our field study did not involve any endangered or protected species.

### Study System

*Achnatherum sibiricum* is a perennial, sparse bunch grass that is native to the Inner Mongolia Steppe of China. It is usually a companion species in the grassland and can sometimes become a dominant species. High incidences of *Neotyphodium* endophyte infection (86–100%) in *A. sibiricum* were recorded in seven native populations in our previous study [30]. In the present study, seeds of *A. sibiricum* were collected from natural population in Hailar in Northeast China (119.67°E, 49.10°N), where the annual mean temperature is around −2°C and annual precipitation about 367 mm. This meadow steppe belongs to a transitional type of habitat between forest and steppe. *Achnatherum sibiricum* within this area is less preferred by mammalian herbivores compared to other dominant species in the community [38]. In the sampled area the dominant species included *Stipa baicalensis* Roshev. and *Leymus chinensis* L., with *A. sibiricum* and *Koeleria cristata* (Linn.) Pers. as common species. Within this population, we collected seeds in August 2008 and stored them at 4°C.

Detection of endophytes using the aniline blue staining method [39] showed that endophyte infection frequency of the Hailar population was 100%. To eliminate the endophyte, we heat treated a subset of randomly chosen seeds in a convection drying oven according to Kannadan and Rudgers [40]. Because disinfection procedures have not been established for this species, we initially treated seeds for 0, 5, 10, 15, 20, 25 or 30 d at 60°C to determine the optimal treatment time. Then all treated seeds were planted in plastic pots filled with vermiculite in November 2008. To assess treatment effectiveness, we examined three leaf peels from each plant under a microscope [39]. In addition, we assessed potential effects of the heat treatment on seed germination and seedling growth. After a 30-d heat treatment, none of the seedlings were infected. Moreover, high temperature treatment had no significant effect on germination rate, germination potential and germination index [41].

### Experimental Design

The plants used in this experiment were cloned from 100 plants grown from seeds that were not heat treated (endophyte-infected, EI) and 100 plants from seeds that were heat treated for 30 d (endophyte-free, EF), multiplied and selected for uniformity in spring of 2009 and 2010. During this period, the plants were clipped repeatedly and kept in vegetative growth. This procedure allowed the subsequent assessment of plant performance to be separated from the initial heat treatment by a round of vegetative reproduction, and is commonly used in endophyte studies [40], [42]. On June 2010, we randomly chose 100 EI and 100 EF tillers (one tiller from each plant), of approximately equal size, and transplanted them evenly into 40 white plastic pots (20 EI and 20 EF pots, five tillers per pot). One pot was 23 cm in diameter and 25 cm in depth and filled with 5 kg of sand. The design of the experiment was completely randomized and a 2×2×2 factorial, with infection status (EI vs. EF), N availability (N+ vs. N−, i.e. supply vs. deficiency), and P availability (P+ vs. P−, i.e. supply vs. deficiency) as the variables. There were five replicates per treatment group. The experiment lasted 49 d, from 5 August to 23 September 2010, and was carried out at the campus experimental field at Nankai University, Tianjin, China. Each ramet (from a single tiller) was examined for endophyte status following staining with lactophenol aniline blue [39] at the end of the experiment.

### Nutrient Treatment

We established four treatments in which nutrient availability was varied, i.e. N+P+, N+P−, N−P+ and N−P−. Ramets from each EI and EF group were grown under all combinations of nutrient availability. The nutrients were supplied by the addition of complete Hoagland nutrient solution. The composition of the nutrient solution was 5.0 mM Ca(NO_3_)_2_, 5.0 mM KNO_3_, 2.5 mM MgSO_4_·7H_2_O, 2.0 mM KH_2_PO_4_, 29 µM Na_2_-EDTA, 20 µM FeSO_4_·7H_2_O, 45 µM H_3_BO_3_, 6.6 µM MnSO_4_, 0.8 µM ZnSO_4_·7H_2_O, 0.6 µM H_2_MoO_4_, 0.4 µM CuSO_4_·5H_2_O and pH 6.0±0.1. For N− treatment, 5.0 mM CaCl_2_ and 5.0 mM KCl were added instead of Ca(NO_3_)_2_ and KNO_3_. For P− treatment, 2.0 mM KCl was added instead of KH_2_PO_4_. The pH was adjusted to 6.0±0.1. During the experiment, 0.8 L of nutrient solution was added twice a week per pot, and 15 times in total. Plants were subjected to ambient light and temperature regimes. The positions of the pots were randomly rotated each week to minimize location effects.

### Growth and Biomass

Measurements of tiller number, leaf number and shoot height of the longest tiller were made on all ramets at the beginning and end of the experiment. At the end of the experiment, leaves, sheaths and roots were harvested separately. Ten fully expanded leaves growing on vegetative tillers per pot were chosen to measure the area and were weighed separately for determination of specific leaf area (SLA). Roots were washed free of soil. Then all plant parts, including leaf blades, sheaths, roots and senesced leaves were separately oven-dried at 60°C.

### Gas Exchange

At the end of the treatments, gas exchange measurements (see below) were made on the youngest fully expanded attached leaf in a pot with a LI-COR 6400 infrared gas analyzer (LI-Cor, Lincoln, NE, USA). The same leaf was also used for measurements of SLA and N content. In this way, differences among leaves of the same plant could be avoided when the relationships among the variables were analyzed.

Photosynthesis-light responses of plants were assessed under 400 µmol mol^−1^ CO_2_. Net photosynthetic rate (Pn) was measured at 1500, 1200, 1000, 800, 500, 300, 200, 150, 100, 50, 20 and 0 µmol m^−2^ s^−1^ PPFD (photosynthetic photon flux density). From the Pn-PPFD curve, Pmax and staturation PPFD were determined.

Photosynthesis-CO_2_ responses of plants were assessed under saturation PPFD, 1200 µmol m^−2^ s^−1^. P_n_ was measured at 1500, 1200, 1000, 800, 600, 400, 300, 200, 150, 100 and 50 µmol mol^−1^ CO_2_ in the reference chamber. The leaf temperature was held constant at 25°C by the equipment. From the P_n_-C_i_ (internal CO_2_ concentration) curve, the parameters needed to calculate the fraction of leaf N allocated to the photosynthetic machinery were determined. The calculation details are as follows.

The P_n_−C_i_ curve was fitted with a linear equation (P_n_ = kC_i_+i) within 50–200 µmol mol^−1^ C_i_ [43]. Maximum carboxylation rate (V_cmax_) and dark respiration rate (R_d_) were calculated according to Farquhar and Sharkey [44] as follows:
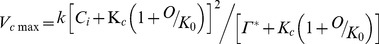


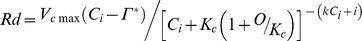
where K_c_ and K_o_ are the Michaelis–Menten constants of Rubisco for carboxylation and oxidation, respectively, and calculated according to Niinemets and Tenhunen [45]. Γ^*^ is the CO_2_ compensation point and O is the intercellular oxygen concentration (close to 210 mmol mol^−1^).

Maximum electron transport rate (J_max_) was calculated according to Loustau et al. [46] as follows:
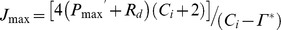
where P_max_′ was determined under saturation PPFD and CO_2_ concentration.

The fractions of total leaf N allocated to carboxylation (P_C_), bioenergetics (P_B_) and light-harvesting (P_L_) of the photosynthetic apparatus were calculated according to Niinemets and Tenhunen [45] as:
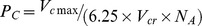


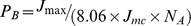


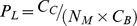
where V_cr_ and J_mc_ are the specific activities of Rubisco and cytochrome f, respectively. N_A_ is total leaf N content, C_C_ is leaf chlorophyll concentration, N_M_ is mass-based leaf N content and C_B_ is ratio of leaf chlorophyll to leaf N in light-harvesting components. The fraction of leaf N allocated to all components of the photosynthetic machinery (P_T_) was calculated as the sum of P_C_, P_B_ and P_L_. Photosynthetic nitrogen use efficiency (PNUE) was calculated as the ratio of P_max_ to area-based leaf N concentration.

### Other Response Variables

The youngest fully expanded leaves were collected for measuring photosynthetic pigment content [47], N and P concentrations. Dried roots were sampled for measuring N and P concentrations. N concentrations of the plant were analyzed using the Kjeldahl method, and P concentrations were measured by molybdenum–antimony colorimetric method [48].

The acid phosphatase secreted by the roots was measured according to the method of Mclanchlan [49]. At harvest, the sand was washed from the roots, water was removed with tissue paper, and 2.0 g of fresh roots (representative sub-sample) were added to sodium acetate-acetic acid buffer with para- nitrophenyl phosphate (PNPP). The concentration of para-nitrophenol in the solution was determined in a spectrophotometer by measuring the absorbance at 405 nm. Phosphatase activity was calculated as the amount of para-nitrophenol produced per g fresh root mass and per hour.

### Statistical Analyses

All statistical analyses were performed with SPSS 10.0 (SPSS, Chicago). For some variables (tiller number, leaf number and biomass allocation), natural log transformation was used to homogenize variance and to obtain a normal distribution of residuals. Effects of N availability, P availability and endophyte infection were analyzed using a three-way analysis of variance (ANOVA). Differences between the means of different treatments and endophyte infection were compared using Duncan's multiple-range tests at P<0.05.

## Results

### Shoot Growth and Biomass Allocation

**Figure 1:**
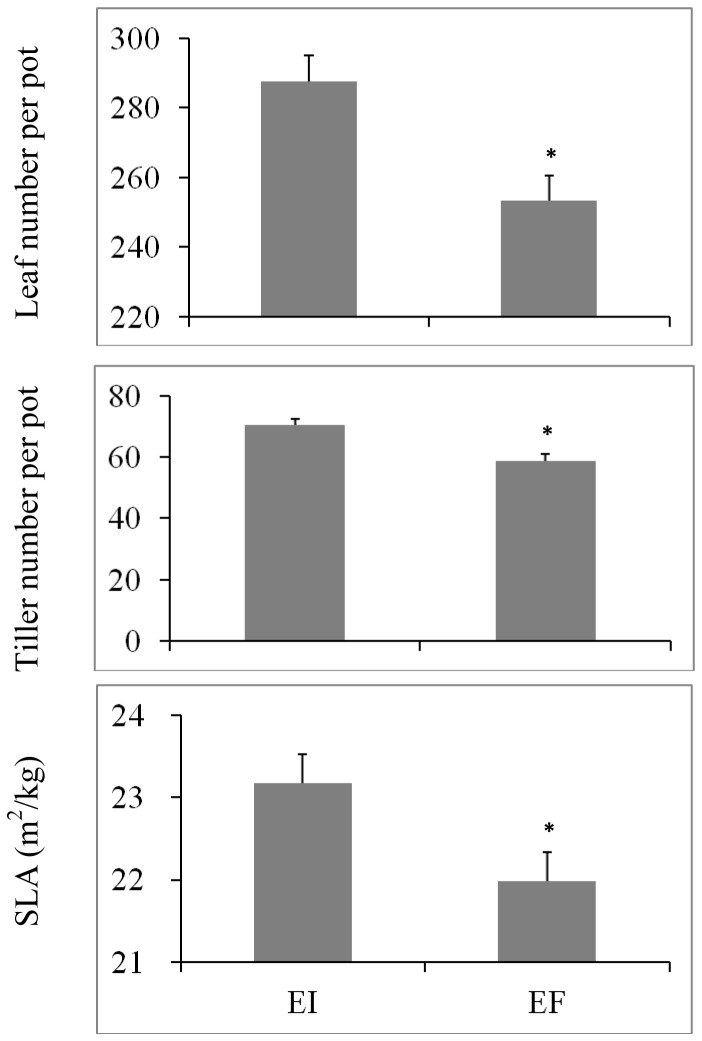
Leaf number, tiller number, and specific leaf area (SLA) of endophyte-infected (EI) or endophyte-free (EF) *Achnatherum sibiricum*. Bars are means+1 SE. Means are data averaged across treatments. An asterisk denotes significance at P<0.05.

At the beginning of the experiment, there were no significant differences between the EI and EF plants in tiller number (*F* = 0.073, *P* = 0.999), leaf number (*F* = 0.279, *P* = 0.958) and shoot height of the longest tiller (*F* = 0.266, *P* = 0.963). Endophyte presence significantly increased tiller number, leaf number and SLA of *A. sibiricum* irrespective of N or P availability (Table 1, Fig. 1). Total biomass was significantly affected by main effects of endophyte status, N and P availability, and the interaction of endophyte ×N×P (Tables 1 and 2). Under N+P− and N−P+ conditions, EI plants had significantly higher shoot, root and total biomass than EF plants. Under N+P+ and N−P− conditions, however, there were no significant differences in biomass between EI and EF plants. Under P+ condition, the biomass of both EI and EF plants decreased with N deficiency; however, the degree of decrease was lower for EI than EF plants. At the same time, both EI and EF plants allocated more resources to roots and thus the root∶shoot ratio increased with N deficiency. Under N+ condition, when compared with P supply, the total biomass of EI plants was maintained with P deficiency; for EF populations, however, the biomass decreased significantly with P deficiency (Table 2).

**Table 1:** Three-way ANOVA for vegetative growth of endophyte-infected (EI) or endophyte-free (EF) *Achnatherum sibiricum*.

		Tiller No.			Leaf No.			SLA			Shoot biomass			Root biomass			Total biomass		
	df	MS	*F*	*P*	MS	*F*	*P*	MS	*F*	*P*	MS	*F*	*P*	MS	*F*	*P*	MS	*F*	*P*
Endophyte (E)	1	1334	13.39	<0.01	11730	10.99	<0.01	53.94	5.426	0.026	28.80	26.58	<0.01	9.120	21.21	<0.01	70.33	31.04	<0.01
Nitrogen (N)	1	8208	82.41	<0.01	61701	57.83	<0.01	88.66	8.917	<0.01	383.2	353.6	<0.01	9.351	21.75	<0.01	512.2	226.1	<0.01
Phosphorus(P)	1	570.0	5.723	0.023	1918	1.798	0.189	13.17	1.324	0.258	87.38	80.65	<0.01	6.956	16.18	<0.01	143.6	63.40	<0.01
E×N	1	7.225	0.073	0.789	24.03	0.023	0.882	0.390	0.039	0.844	0.445	0.411	0.526	0.004	0.008	0.928	0.529	0.233	0.632
E×P	1	42.03	0.422	0.521	1113	1.043	0.315	10.44	1.050	0.313	0.702	0.648	0.427	0.180	0.418	0.523	1.592	0.703	0.408
N×P	1	207.0	2.079	0.159	235.2	0.220	0.642	4.323	0.435	0.514	3.091	2.853	0.101	1.616	3.759	0.061	9.178	4.051	0.053
E×N×P	1	286.2	2.874	0.100	60.03	0.056	0.814	0.206	0.021	0.886	11.13	10.27	<0.01	1.552	3.611	0.066	20.99	9.267	<0.01
Residual	32	99.60			1067			9.942			1.083			0.430			2.266		

**Table 2:** Biomass allocation of endophyte-infected (EI) or endophyte-free (EF) *Achnatherum sibiricum* under various conditions of N and P availability.

Treatment			Shoot biomass (g)	Root biomass(g)	Total biomass(g)	Root∶ Shoot
P+	N+	EI	14.15±1.299a	5.14±0.846a	19.30±1.999a	0.36±0.042c
		EF	13.56±1.501a	4.70±0.691a	18.26±1.929a	0.35±0.049c
	N−	EI	9.36±0.775b	4.95±0.428a	14.32±1.088b	0.53±0.042 b
		EF	7.09±0.579c	3.76±0.437b	10.85±0.790c	0.53±0.069 b
P−	N+	EI	13.07±1.271a	5.24±0.547a	18.31±1.748a	0.40±0.024c
		EF	9.84±1.247b	3.74±0.915b	13.58±1.897b	0.38±0.078c
	N−	EI	5.06±0.424d	3.46±0.494bc	8.52±0.446d	0.69±0.142a
		EF	4.37±0.672d	2.78±0.701c	7.15±1.332d	0.63±0.088a

Note. Values are means ± SE. Significant differences (*P*<0.05) for each variable are indicated by lowercase letters for variables where N, P availability and endophyte infection were analyzed together.

### N Allocation and Photosynthesis

Area-based leaf N content (N_A_) was significantly affected by endophyte infection, N and P availability as well as their interaction (Table 3). In all treatments, N_A_ of EI was lower than that of EF plants (Table 4). N_A_ of EI was significantly affected by N supply but not by P supply. For N_A_ of EF, however, it was significantly affected by both N and P supply. When N allocation was considered, there were differences between EI and EF plants and/or among different treatments. With N supply, EI plants had similar or slightly higher N fractions allocated to the photosynthetic machinery (P_T_) when compared with their EF counterparts. With N deficiency, however, the above N fraction in EI plants was significantly higher compared to their EF counterparts.

**Table 3:** Three-way ANOVA for photosynthetic parameters, N allocation and acid phosphatase activity of endophyte-infected (EI) or uninfected (EF) ramets of *Achnatherum sibiricum*.

		N_A_			P_max_			PNUE			P_T_			Acid phosphatase activity		
	df	MS	*F*	*P*	MS	*F*	*P*	MS	*F*	*P*	MS	*F*	*P*	MS	*F*	*P*
Endophyte (E)	1	0.567	90.06	<0.01	31.91	17.67	<0.01	895.2	320.7	<0.01	1.108	83.08	<0.01	0.003	18.35	<0.01
Nitrogen (N)	1	4.332	687.6	<0.01	215.1	119.1	<0.01	771.8	276.5	<0.01	1.396	104.7	<0.01	0.029	185.1	<0.01
Phosphorus(P)	1	0.050	7.945	<0.01	3.869	2.142	0.156	18.99	6.803	0.015	0.035	2.628	0.125	0.005	30.73	<0.01
E×N	1	0.001	0.080	0.780	5.946	3.292	0.082	209.3	74.97	<0.01	0.434	32.56	<0.01	0.000	1.587	0.217
E×P	1	0.045	7.112	0.012	4.095	2.267	0.145	19.26	6.901	0.015	0.000	0.015	0.905	0.000	1.587	0.217
N×P	1	0.017	2.646	0.114	0.581	0.322	0.576	21.24	7.609	0.011	0.118	8.816	<0.01	0.021	134.3	<0.01
E×N×P	1	0.030	4.688	0.038	5.115	2.832	0.105	0.647	0.232	0.635	0.027	1.996	0.177	0.000	1.587	0.217
Residual	32	0.006			1.806			2.791			0.013			0.000		

Note. N_A_, total leaf nitrogen content; P_max_, maximum net photosynthetic rate; PNUE, photosynthetic nitrogen use efficiency; P_T_ the fraction of leaf nitrogen allocated to all components of the photosynthetic machinery.

**Table 4:** N allocation and maximum photosynthetic rate of endophyte-infected (EI) or endophyte-free (EF) *Achnatherum sibiricum* under various conditions of N and P availability.

Treatment			N_A_	P_T_	P_max_
P+	N+	EI	0.92±0.084c	0.719±0.059c	15.58±1.323a
		EF	1.28±0.100a	0.474±0.034d	12.80±1.068b
	N−	EI	0.35±0.036f	1.240±0.227b	10.60±0.708c
		EF	0.61±0.081d	0.614±0.124cd	7.95±0.844d
P−	N+	EI	1.02±0.114c	0.571±0.037cd	15.23±1.223a
		EF	1.13±0.080b	0.471±0.051d	12.29±2.773bc
	N−	EI	0.25±0.075f	1.529±0.116a	8.12±0.463d
		EF	0.49±0.023e	0.758±0.139c	8.50±0.972d

Note. N_A_, total leaf nitrogen content in g m^−2^; P_T_, the fraction of leaf nitrogen allocated to all components of the photosynthetic machinery in g g^−1^; P_max_, maximum net photosynthetic rate in µmol m^−2^ s^−1^. Values are means ± SE. Significant differences (*P*<0.05) for each variable are indicated by lowercase letters for variables N, P availability and endophyte infection were analyzed together.

**Figure 2:**
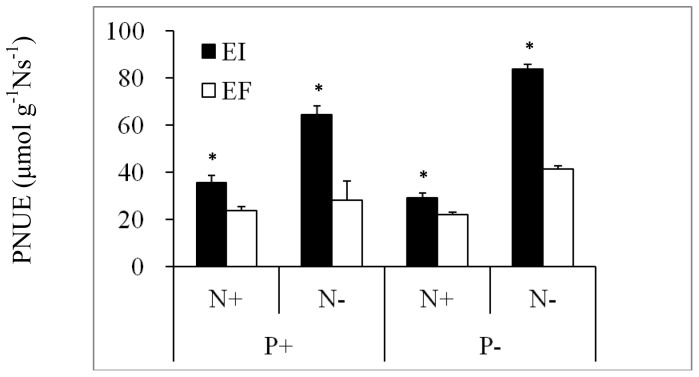
Photosynthetic nitrogen use efficiency (PNUE) of endophyte-infected (EI) or endophyte-free (EF) *Achnatherum sibiricum* under various conditions of N and P availability. Bars are means+1 SE. An asterisk denotes significance at P<0.05.

The maximum net photosynthetic rate for EI tended to be higher than that of EF, but there was a significant difference only in N+P− and N−P+ treatments (Table 4). When PNUE was considered, it was significantly higher for EI compared to EF plants in all treatments (Fig. 2).

### Acid Phosphatase Activity

**Figure 3:**
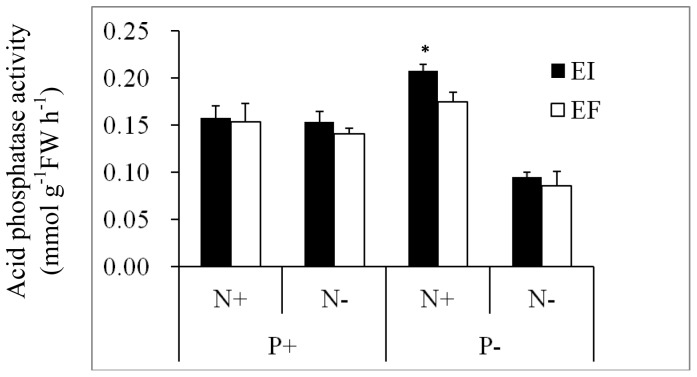
Acid phosphatase activity of endophyte-infected (EI) or endophyte-free (EF) *Achnatherum sibiricum* under various conditions of N and P availability. Bars are means+1 SE. An asterisk denotes significance at P<0.05.

Acid phosphatase activity was significantly affected by main effects of endophyte status, N and P availability and the interaction N×P (Table 3). In all treatments, the acid phosphatase activity of EI plants tended to be higher than that of EF plants, but the difference was significant only in N+P− treatment (Fig. 3).

### Plant N and P Concentrations

Both leaf and root N and P concentrations of *A. sibiricum* were significantly affected by N and P availability as well as endophyte infection (Table 5). Leaf N concentration was significantly lower for EI compared to EF plants. Under N+ conditions, EI leaf N concentration was not affected by P deficiency, while EF leaf N decreased significantly with P deficiency. Under P+ conditions, both EI and EF leaf P concentrations decreased significantly with N deficiency. Endophyte infection had no effect on leaf P concentration but significantly increased root P concentration. Total N concentration (N concentration of the whole plant) was significantly decreased, while total P concentration (P concentration of the whole plant) was significantly increased by endophyte infection, and thus N∶P ratio was significantly lower for EI compared to EF plants (Table 6).

**Table 5:** Three-way ANOVA for ecological stoichiometry of endophyte-infected (EI) or endophyte-free (EF) ramets of *Achnatherum sibiricum*.

		N concentration									P concentration								
		Leaf			Root			Total			Leaf			Root			Total		
	df	MS	*F*	*P*	MS	*F*	*P*	MS	*F*	*P*	MS	*F*	*P*	MS	*F*	*P*	MS	*F*	*P*
Endophyte (E)	1	196.7	141.6	<0.01	13.47	8.609	<0.01	34.56	68.19	<0.01	0.456	3.617	0.066	7.639	165.8	<0.01	1.845	31.37	<0.01
Nitrogen (N)	1	2563	1845	<0.01	579.5	370.4	<0.01	1688	3331	<0.01	2.793	22.16	<0.01	2.266	49.18	<0.01	1.914	32.55	<0.01
Phosphorus(P)	1	33.62	24.20	<0.01	14.92	9.538	<0.01	41.01	80.92	<0.01	7.048	55.92	<0.01	0.471	10.22	<0.01	3.209	54.57	<0.01
E×N	1	2.084	1.500	0.230	0.034	0.022	0.883	0.600	1.184	0.285	0.006	0.048	0.829	3.919	85.05	<0.01	0.460	7.823	<0.01
E×P	1	13.70	9.864	<0.01	27.41	17.52	<0.01	1.406	2.775	0.106	0.215	1.703	0.201	0.119	2.579	0.118	0.065	1.102	0.302
N×P	1	8.363	6.021	0.020	2.475	1.582	0.218	0.204	0.404	0.530	5.206	41.31	<0.01	0.870	18.89	<0.01	1.853	31.51	<0.01
E×N×P	1	12.92	9.300	<0.01	42.42	27.11	<0.01	0.001	0.002	0.961	0.047	0.372	0.546	0.004	0.096	0.759	0.044	0.752	0.392
Residual	32	1.389			1.564			0.507			0.126			0.046			0.059		

**Table 6:** N and P concentration of endophyte-infected (EI) or endophyte-free (EF) *Achnatherum sibiricum* under various conditions of N and P availability.

Treatment			N concentration (g/kg)			P concentration (g/kg)			N∶P ratio		
			Leaf	Root	Total	Leaf	Root	Total	Leaf	Root	Total
P+	N+	EI	22.61±0.325c	18.55±1.255a	20.61±0.331b	4.40±0.304a	2.28±0.145cd	3.61±0.203a	5.16±0.334c	8.15±0.845a	5.72±0.331c
		EF	28.89±1.559a	13.62±1.712c	23.08±0.688a	3.99±0.259a	2.12±0.265d	3.25±0.168b	7.27±0.778b	6.52±1.364b	7.11±0.452b
	N−	EI	8.19±0.717f	8.32±1.240d	8.00±0.412e	3.10±0.433b	2.70±0.125ab	2.89±0.193c	2.71±0.665ef	3.09±0.522d	2.77±0.216ef
		EF	13.12±1.215d	7.63±1.410de	10.00±0.913d	2.79±0.426b	1.34±0.190e	2.24±0.374ef	4.77±0.630cd	5.82±1.439b	4.58±0.920d
P−	N+	EI	24.00±1.585c	13.11±0.780c	19.10±0.922c	2.62±0.237b	2.88±0.205a	2.47±0.091de	9.19±0.715a	4.57±0.454c	7.74±0.408b
		EF	25.66±1.338b	15.62±0.833b	20.84±0.805b	2.65±0.518b	2.55±0.391bc	2.40±0.403ef	9.97±1.830a	6.24±0.950b	8.86±1.371a
	N−	EI	5.48±1.409g	8.00±1.258de	6.21±0.713f	2.91±0.229b	2.76±0.132ab	2.74±0.139cd	1.88±0.450f	2.89±0.370d	2.26±0.161f
		EF	10.33±0.531e	6.50±1.258e	7.44±0.685e	2.750.313b	1.13±0.125e	2.12±0.187f	3.80±0.470de	5.73±0.602bc	3.53±0.290e

Note. Values are means ± SE. Significant differences (*P*<0.05) for each variable are indicated by lowercase letters for variables N, P availability and endophyte infection were analyzed together.

## Discussion

The present study demonstrated that a beneficial interaction between the native grass *A. sibiricum*, and its associated fungal endophyte (*Neotyphodium* sp.) depended on both N and P availability. When only N or P was limited, EI plants accumulated significantly more aboveground biomass and total biomass than EF plants. When both N and P were limited, however, the benefits of endophyte infection declined. These findings are in agreement with reports on the response of perennial ryegrass to N deficiency [18] and tall fescue to P deficiency [24], in which only one element (N or P) was deficient; and also in agreement with previous research that EI plants have no advantage over EF plants at low nutrient availabilities [5], [8], [50]. We did not find a significant advantage of EI plants over their EF counterparts when N and P were supplied – this has been reported previously in tall fescue [8]. A possible explanation for this difference is that the nutrients in the medium where *A. sibiricum* grew were not sufficiently high. McCormick et al. [4] also found EI *Danthonia spicata* did not have a performance advantage relative to EF plants under fertilized conditions, in which the medium where *D. spicata* grew was extremely nutrient poor even in the fertilized treatment. Overall, our results suggested that the benefits from endophyte infection depended largely on the supply of N and/or P. When both N and P were limited simultaneously, the benefits from endophyte infection disappeared.

When N was supplied, EI plants had similar N concentration and total biomass regardless of P status, while EF plants had significantly lower N and total biomass in P deficiency compared to P supply. There were no significant differences in P concentration between shoots of EI and EF plants in all treatments. This suggests that the beneficial effect of endophyte infection on biomass production of the host plants was more strongly regulated by the availability of N rather than P [51]. With N supply, endophyte infection may help the host grass in maintaining biomass regardless of P status. With N deficiency, even with P supply, the biomass of both EI and EF plants decreased; however, EI biomass decreased slowly.

Current knowledge suggests that leaf N content is correlated with photosynthetic capacity [52]. In the present experiment, N concentration was lower for EI compared to EF plants; however, EI plants allocated significantly higher fractions of N to photosynthetic machinery with N deficiency. EI plants had significantly lower leaf N concentration but significantly higher maximum photosynthetic rate, PNUE and total biomass than did EF plants in the N−P+ treatment. It has been reported that organisms with a greater growth advantage in nutrient-poor environments are those able to modify their body nutrient content and increase efficiency of nutrient use without major decreases in their growth rates [53]–[54]. Under N−P+ conditions, EI plants grew better than EF plants by lowering their N concentration while increasing their N allocation to photosynthetic machinery. Therefore, it is N allocation to photosynthetic machinery instead of leaf N concentration itself that was more highly correlated with plant growth [55]–[56].

In N+P− treatment, P concentration in the shoot of EI and EF plants was similar but EI roots had significantly higher P concentration than EF roots, and similar results were reported by Zabalgogeazcoa et al. [57] on the response of *Festuca rubra* grown in low nutrient soil. Higher root P concentration here was attributed to higher acid phosphatase activity of EI roots. Phosphatase is an enzyme excreted by plant roots, fungi and bacteria and may contribute to as much as 65% of the annual P uptake of grasses [58]. A series of studies have shown that phosphatase activity was increased by AM fungal colonization [59]. Thus, was high acid phosphatase activity of EI roots related to AM colonization? Endophytes in grasses have been reported to reduce mycorrhizal colonization of host roots as well as spore densities in the soil [60]–[61]. In our sampled area in the Inner Mongolia Steppe, Bao [62] found that AM infected over 80% of Gramineae; however, the average infection rate was relatively low (i.e. about 28%). AM infection was not found in the *Achnatherum* genus. In the present study, although we did not measure AM colonization of the roots, the plants were grown from seeds collected in the natural grassland where AM colonization was not found in the *Achnatherum* genus, so it is reasonable to assume they were not colonized by mycorrhizae. Therefore, in the N+P− treatment in the present study, it is endophyte infection that significantly improved acid phosphatase activity of the host grass, which led to higher root P concentration and further higher total biomass in EI compared to EF plants.

The results presented here agreed with the initial prediction that beneficial interaction between the native grass *A. sibiricum* and its associated fungal endophyte depended on both N and P availability. The results further suggested that the beneficial effect of endophyte infection was more conditional on N than P. Under N+P− conditions, endophyte infection significantly improved acid phosphatase activity of EI plants, and so biomass of EI plants was not affected by P deficiency, and resulted in a greater P concentration and more biomass in EI than EF plants. Under N−P+ conditions, both EI and EF biomass decreased compared with N+P+ conditions. EI plants had decreased leaf N concentration but allocated higher fractions of N to photosynthetic machinery compared to EF plants, which resulted in a slow decrease of EI growth– thus EI plants had significantly more biomass than EF plants. Under N−P− conditions, EI plants allocated higher fractions of N to photosynthesis and had a greater P concentration in roots, but there was no significant difference in biomass between EI and EF plants. Additionally, we did not find a clear cost of endophyte infection even in the N−P− treatment. Admittedly, the duration of the field pot experiment was short in comparison with the natural life span of the grass host and our results should be interpreted with caution. We propose that future studies should examine a wider range of native grass-endophyte systems in long-term field studies to better understand the general role of defensive mutualism in endophyte-plant interactions.
